# A Meta-Analysis of Retinoblastoma Copy Numbers Refines the List of Possible Driver Genes Involved in Tumor Progression

**DOI:** 10.1371/journal.pone.0153323

**Published:** 2016-04-26

**Authors:** Irsan E. Kooi, Berber M. Mol, Maarten P. G. Massink, Marcus C. de Jong, Pim de Graaf, Paul van der Valk, Hanne Meijers-Heijboer, Gertjan J. L. Kaspers, Annette C. Moll, Hein te Riele, Jacqueline Cloos, Josephine C. Dorsman

**Affiliations:** 1 Department of Clinical Genetics, VU University Medical Center, Amsterdam, The Netherlands; 2 Department of Bio-medical Genetics, University Medical center Utrecht, Utrecht, The Netherlands; 3 Department of Radiology and Nuclear Medicine, VU University Medical Center, Amsterdam, The Netherlands; 4 Department of Pathology, VU University Medical Center, Amsterdam, The Netherlands; 5 Department of Pediatric Oncology/Hematology, VU University Medical Center, Amsterdam, The Netherlands; 6 Department of Ophthalmology, VU University Medical Center, Amsterdam, the Netherlands; 7 Division of Biological Stress Response, Netherlands Cancer Institute, Amsterdam, The Netherlands; 8 Department of Hematology, VU University Medical Center, Amsterdam, The Netherlands; University of Texas MD Anderson Cancer Center, UNITED STATES

## Abstract

**Background:**

While *RB1* loss initiates retinoblastoma development, additional somatic copy number alterations (SCNAs) can drive tumor progression. Although SCNAs have been identified with good concordance between studies at a cytoband resolution, accurate identification of single genes for all recurrent SCNAs is still challenging. This study presents a comprehensive meta-analysis of genome-wide SCNAs integrated with gene expression profiling data, narrowing down the list of plausible retinoblastoma driver genes.

**Methods:**

We performed SCNA profiling of 45 primary retinoblastoma samples and eight retinoblastoma cell lines by high-resolution microarrays. We combined our data with genomic, clinical and histopathological data of ten published genome-wide SCNA studies, which strongly enhanced the power of our analyses (N = 310).

**Results:**

Comprehensive recurrence analysis of SCNAs in all studies integrated with gene expression data allowed us to reduce candidate gene lists for 1q, 2p, 6p, 7q and 13q to a limited gene set. Besides the well-established driver genes *RB1* (13q-loss) and *MYCN* (2p-gain) we identified *CRB1* and *NEK7* (1q-gain), *SOX4* (6p-gain) and *NUP205* (7q-gain) as novel retinoblastoma driver candidates. Depending on the sample subset and algorithms used, alternative candidates were identified including *MIR181* (1q-gain) and *DEK* (6p gain). Remarkably, our study showed that copy number gains rarely exceeded change of one copy, even in pure tumor samples with 100% homozygosity at the *RB1* locus (N = 34), which is indicative for intra-tumor heterogeneity. In addition, profound between-tumor variability was observed that was associated with age at diagnosis and differentiation grades.

**Interpretation:**

Since focal alterations at commonly altered chromosome regions were rare except for 2p24.3 (*MYCN*), further functional validation of the oncogenic potential of the described candidate genes is now required. For further investigations, our study provides a refined and revised set of candidate retinoblastoma driver genes.

## Introduction

Retinoblastoma is a pediatric cancer of the retina. Although the disease is relatively rare accounting for 2% of childhood cancers [1], retinoblastoma is the most common intra-ocular malignancy in children [2]. Retinoblastoma development is initiated by two sequential hits [3] of *RB1* (*RB1*^-/-^ patients) and in few cases by amplification of *MYCN* (*RB1*^*+/+*^*MYCN*^*A*^ patients) [4]. Hereditary patients carry a deleterious germ line mutation in one *RB1* allele and therefore only require one somatic mutation in the wild type *RB1* allele for retinoblastoma to develop while non-hereditary patients require two somatic mutations in *RB1*. However, while bi-allelic inactivation of *RB1* can cause benign retinoma lesions, additional genetic alterations can be required for progression to retinoblastoma [5].

Several studies aimed at identifying genetic alterations subsequent to *RB1* inactivation have yielded useful insights. Early comparative genome hybridization (CGH) [6–10], array-CGH [11–13] and more recent single nucleotide polymorphism (SNP)-array profiling studies [14–16] showed that retinoblastoma genomes frequently (>10%) contain somatic copy number alterations (SCNAs) including gains at chromosomal arms 1q, 2p, 6p, 13q and 19q and losses at 13q, 16q and 17p. Except for the 2p-arm (2p24.3, *MYCN*), focal SCNAs (< 3 Mb) are rare [15] complicating the refinement of minimal regions of gain/loss (MR[G/L]) to a single gene level. Moreover, sample sizes of published studies were small which further impeded identification of driver genes within recurrent SCNA-regions.

In addition, it is has been demonstrated that there is profound variability in the total amount of genomic disruption by SCNAs between retinoblastoma tumors [15]. In several studies it was discussed whether and how the extent of genomic disruption relates to clinical and histopathological variables. However, due to strong connectivity between the independent variables (like age at diagnosis, heredity, laterality and differentiation) and small sample sizes, explanations for the variability in genomic disruption in retinoblastoma were inconclusive due to limited power.

Our study aims to refine the set of putative driver genes of recurrent SCNAs and get insight into the variability in genomic disruption. Data from high-resolution genome-wide SNP-arrays of 45 human retinoblastoma samples matched with peripheral blood DNA were used and complemented with clinical and histopathological features. In order to increase the power of our study, results were analyzed together with results of ten published genome-wide SCNA-profiling retinoblastoma studies [6–11,13,15–17] adding up to a considerable number of 310 primary retinoblastoma samples.

## Results

### Good agreement in SCNA-frequencies between 11 independent studies

**Fig 1:**
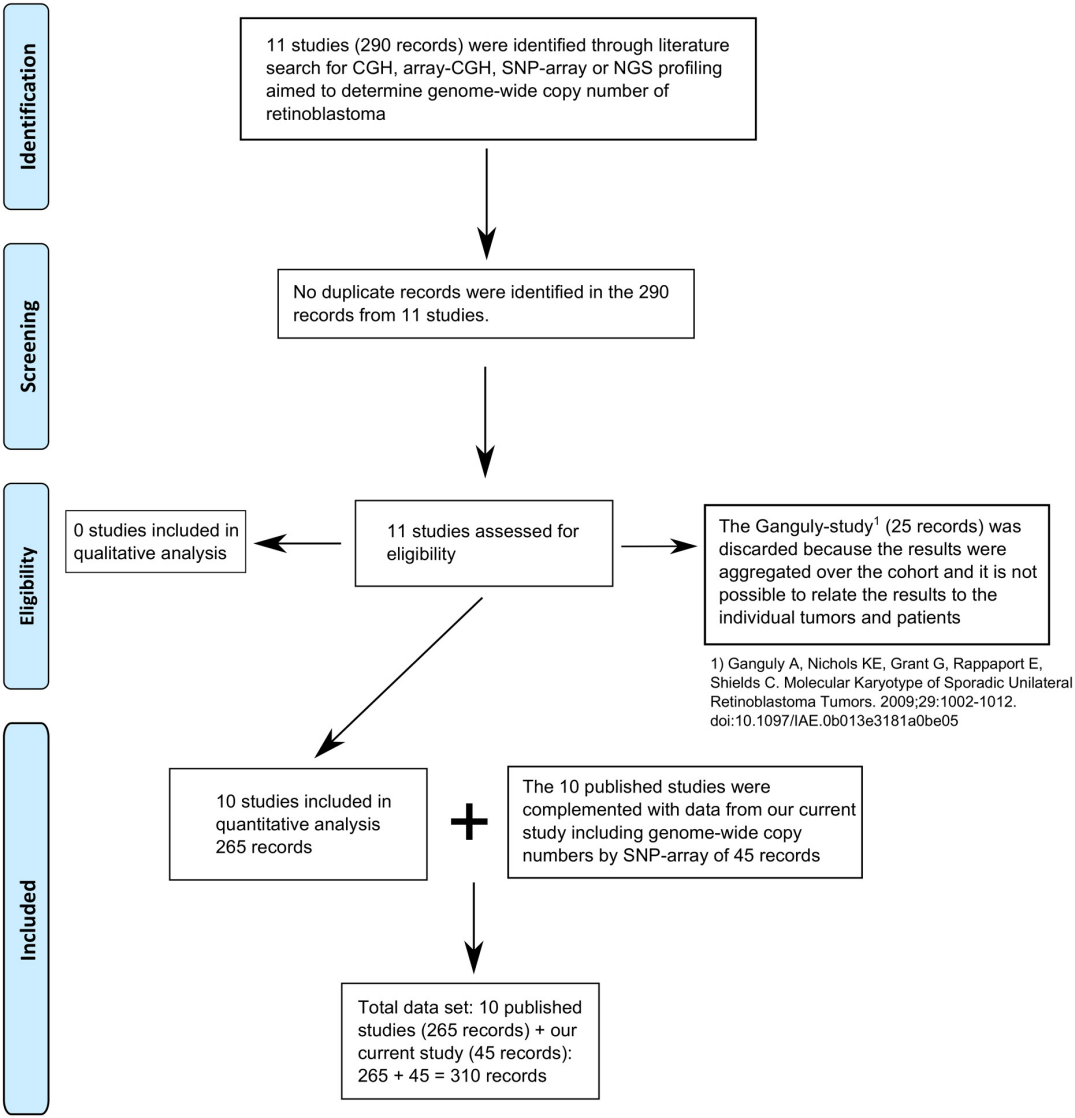
PRISMA flow chart. Searching google scholar for “retinoblastoma”, “copy number”, “(a)CGH”, and “SNP-array”, 11 studies were identified that performed genome-wide profiling of retinoblastoma adding up to 290 samples. No duplicates samples were identified. For the Ganguly study [14], copy number results could not be linked to individual tumors and therefore this study, including 25 samples was discarded. The remaining 265 samples were all included in quantitative analysis and were complemented with 45 SNP-arrays from our current study, adding up to 310 samples.

**Fig 2:**
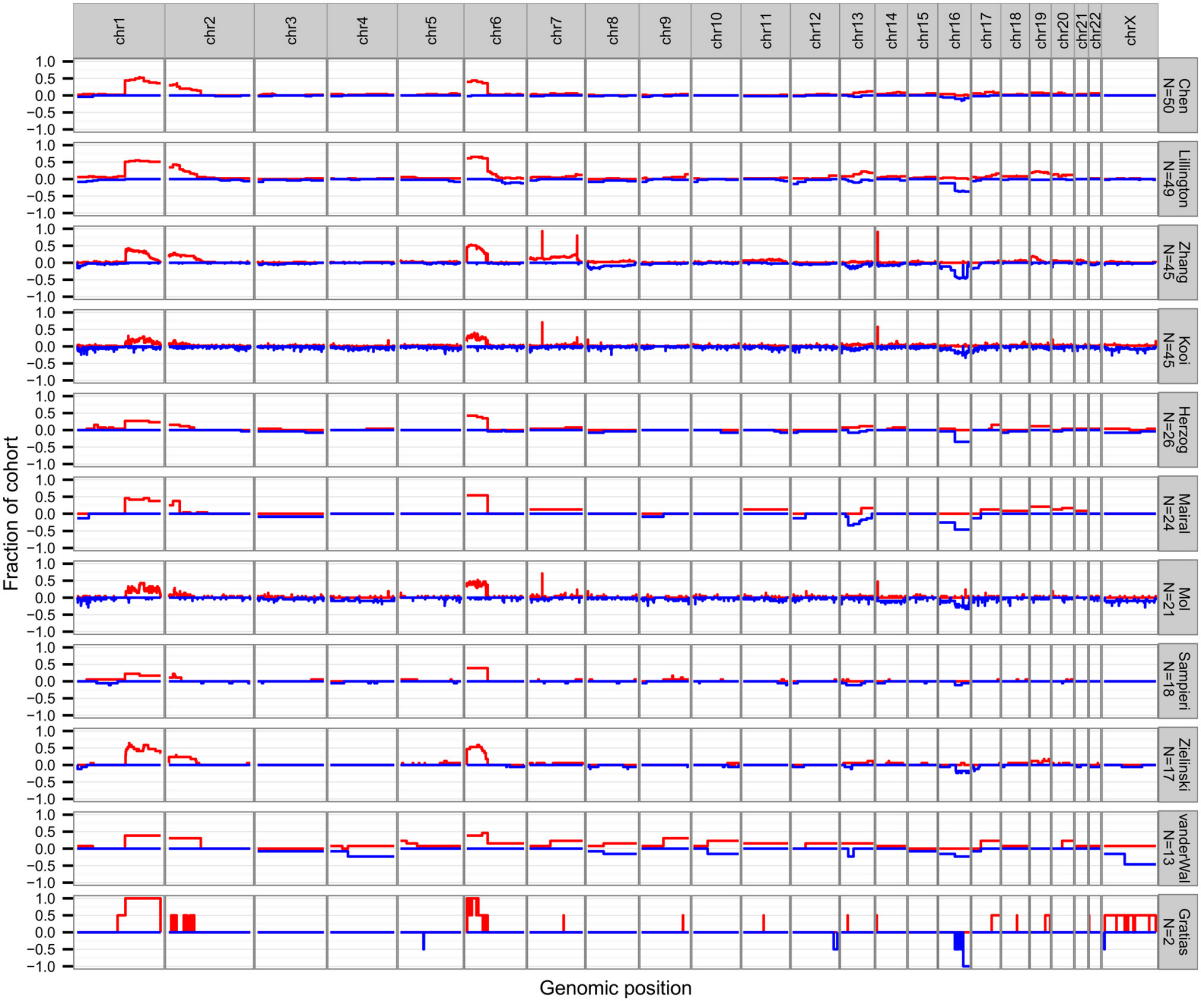
SCNA frequencies per study. Genome-wide SCNA-frequencies (DNA copy number gains in red, losses in blue) detected in primary retinoblastoma samples in our current study (Kooi, N = 45) and in ten published studies. Studies are ordered by sample size where the largest study is placed at the top.

Our study describes SCNAs of 45 primary retinoblastoma samples together with SCNAs from 10 published studies (S1 Table) adding up to 310 tumor samples (Fig 1, cohort description is given S2 Table). This allowed for a detailed genome-wide comparison of SCNAs determined from independent and international studies. For each HGNC approved gene, the percentage of the cohort affected by gains/losses is visualized along the genomic coordinates stratified by study (Fig 2). Percentages of SCNAs showed good agreement between studies, except for studies with small sample size and/or platform related differences. In the two small-sized studies (Gratias; N = 2, van der Wal, N = 13), SCNA percentages more easily reached high numbers. For example, in the Gratias study [13], gain of chromosome 1 and 6 was observed in 100% of the cohort, yet only affecting two patients. In addition, some platform specific differences between studies were reflected in the SCNA-percentages. For example, high-resolution studies (SNP-arrays, Kooi, Mol, Zhang) were able to detect small (< 100 Kb) SCNAs which resulted in more spikes in SCNA-frequencies. Three notable SCNA-gain spikes at 7p14.1, 7q34 and 14q11.2 overlapping with the TCR-γ, TCR-β and TCR-α/δ gene clusters respectively, likely were not cancer related. It was shown that loss of these three regions is a frequent and somatic event that occurs in lymphocytes [18]. As a result, the three spikes of SCNA-gains appeared in the tumor-blood matched SNP-array analyses. Most likely these gains were the result of lymphocyte specific deletions and therefore the TCR-α/β/γ/δ genes were omitted from further analyses in this study.

### Identification of candidate retinoblastoma driver genes

A major challenge in the interpretation of SCNAs is to distinguish driver from passenger genes since a single SCNA usually covers tens to hundreds of genes. To do so, SCNA-gain and SCNA-loss frequencies were used to compile a list of most plausible candidate genes and were integrated with micro-array gene expression data. For each approved HGNC gene, the number of patients with SCNA-gains subtracted from the number of patients with losses was calculated, which we call the SCNA-gain-loss difference. By subtracting the number of patients with gene losses from the number of patients with gene gains, non-disease associated SCNAs that arose from random genomic instability, which usually are both gained and lost in a cohort, were not prioritized. In addition to the SCNA-gain-loss difference, the percentage of patients affected by gain/loss of the respective gene (SCNA-percentage) was used as an extra threshold for candidate gene selection.

**Fig 3:**
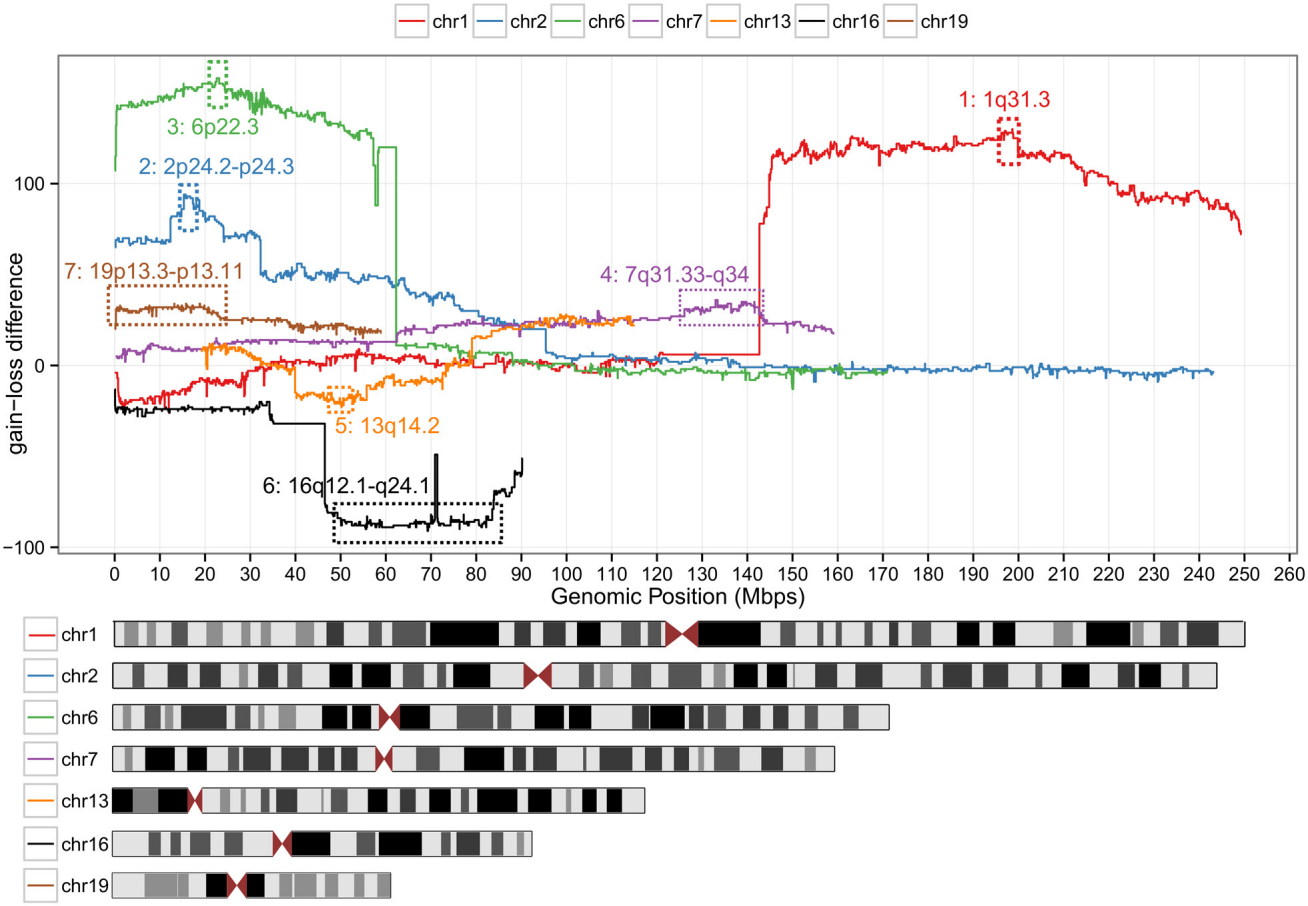
Identification of SCNA peak regions. For chromosomes that contained commonly altered genes, the gain-loss difference (number of patients with gains minus the number of patients with losses) is plotted for each official HGNC along chromosomal coordinates. For each of these chromosomes, peak regions were defined (also see S3 Table) indicated by the dashed rectangles and were used for retinoblastoma driver discovery.

We considered genes with an SCNA-percentage >10% (out of 310 retinoblastoma samples) as candidate genes. This frequency threshold was empirically determined by plotting the number of genes that meet increasing SCNA percentage criteria (S1 Fig). This figure shows that the number of genes passing the SCNA percentage filter decreases rapidly but stabilizes at SCNA percentage between 5 and 15%. Chromosomes that contained candidate genes included chromosomes 1, 2, 6, 7, 13, 16 and 19. For these chromosomes, the SCNA-gain-loss difference is visualized along genomic coordinates (Fig 3). For each chromosome, peaks were defined by the gene with the highest SCNA-gain-loss difference and neighboring genes that showed at maximum 1% decrease in SCNA-gain-loss difference relative to the peak gene, visualized by the dashed numbered rectangles (Fig 3). Genomic coordinates with annotated gene symbols are given (S3 Table). For the Mol study [15] and our current study, matching gene expression profiling was available for 56 samples (S4 Table) [19]. Using this dataset, gene dosage effects (more/less gene copies is correlated with more/less gene expression) were quantified. For each peak region, genes with a significant gene dosage effect are listed in the last column of S3 Table. Whereas the candidate list for chromosomes 1, 2, 6, 7 and 13 (relatively small peak regions) was narrowed down to a handful of genes, for chromosomes 16 and 19 (relatively large peak regions) there were still many candidate genes remaining. In S5 Table, more detailed information is given on the candidate genes including the mean expression of the respective gene in the expression profiling cohort. For candidate genes on chromosome 1, 2, 6, and 7, genes with the highest mean expression were *NEK7*, *MYCN* and *DDX1*, *E2F3* and *SOX4*, and *CHCHD3* respectively. For chromosomes 13 containing deletions, *RB1* had the lowest mean expression. For candidates on chromosomes 16 and 19 there were no genes that clearly showed the lowest or highest expression. Therefore, narrowing down retinoblastoma-associated genes any further than presented in S3 Table was considered too speculative and was therefore omitted for chromosomes 16 and 19.

To test the robustness of the peaks identified by the described meta-analysis (S3 Table) and to identify smaller or additional peak regions, subset analyses were performed on the high-resolution SNP-array studies for which raw data was available (Mol, Zhang, Kooi, N = 111). In addition to DNAcopy (circular binary segmentation), genoCN (Hidden Markov-Model) was used to infer the copy number states gain, loss and unchanged. The resulting gene-wise frequencies of gain and loss (S2 Fig) are visualized per study. The gain-loss differences determined by genoCN were strongly correlated (correlation test p-value < 2.2E-16, r = 0.95) with the gain-loss differences determined with DNAcopy (S5 Table). Yet for 1q and 6p, the gain-loss differences determined by genoCN reached a maximum at slightly different genes; for 1q at *MIR181* and for 6p at *KDM1B*, *DEK*, *RNA6-263P*, *RNF144B* and *MIR548A1*(S5 Table). The *DEK* gene displayed the most significant gene-dosage effect (FDR-adjusted p-value 3.25E-05) and is one of the most highly expressed genes interrogated by the micro-array (rank 38/18,290). In addition to genoCN segmentation, GISTIC analysis was performed on the Mol, Zhang and Kooi subset (S3 Fig, S5 and S6 Tables). This algorithm uses not only SCNA frequency and recurrence but also the SCNA amplitude to identify significantly altered regions. GISTIC analysis confirmed the significance of arm-level gains at 1q and 6p and loss at 13q and 16q and also confirmed peak 2 (*MYCN*, 2p24.3), which was identified by the meta-analysis. While GISTIC analysis could not identify a clear focal peak at 6p, multiple peaks were identified at 1q and 13q, signifying the difficulty of single-gene identification. The highest peak at 1q in GISTIC analysis at 1q32.1 included 39 genes, but did not overlap with peak 1 identified by the meta-analysis (S3 Table). The GISTIC peaks at 13q also did neither overlap with corresponding peak 5 (13q) from the meta-analysis nor with the *RB1* locus. Additionally to the peaks identified by the meta-analysis (S3 Table), GISTIC identified focal gain of 14q22.3 including *OTX2* (gene dosage effect FDR-adjusted p-value 0.07) also discussed in the Mol study, loss of 1p36.32 (138 genes, S5 and S6 Tables) and loss of 17p13.3 (80 genes, S5 and S6 Tables). Of note, the GISTIC gain peaks at 7p14.1, 7q34 and 14q11.2 are the regions that are commonly lost in lymphocyte DNA, not gained by retinoblastomas (also see section 4.1, Fig 2) [18].

### Clustering of tumors based on SCNAs is mainly driven by total genomic disruption

**Fig 4:**
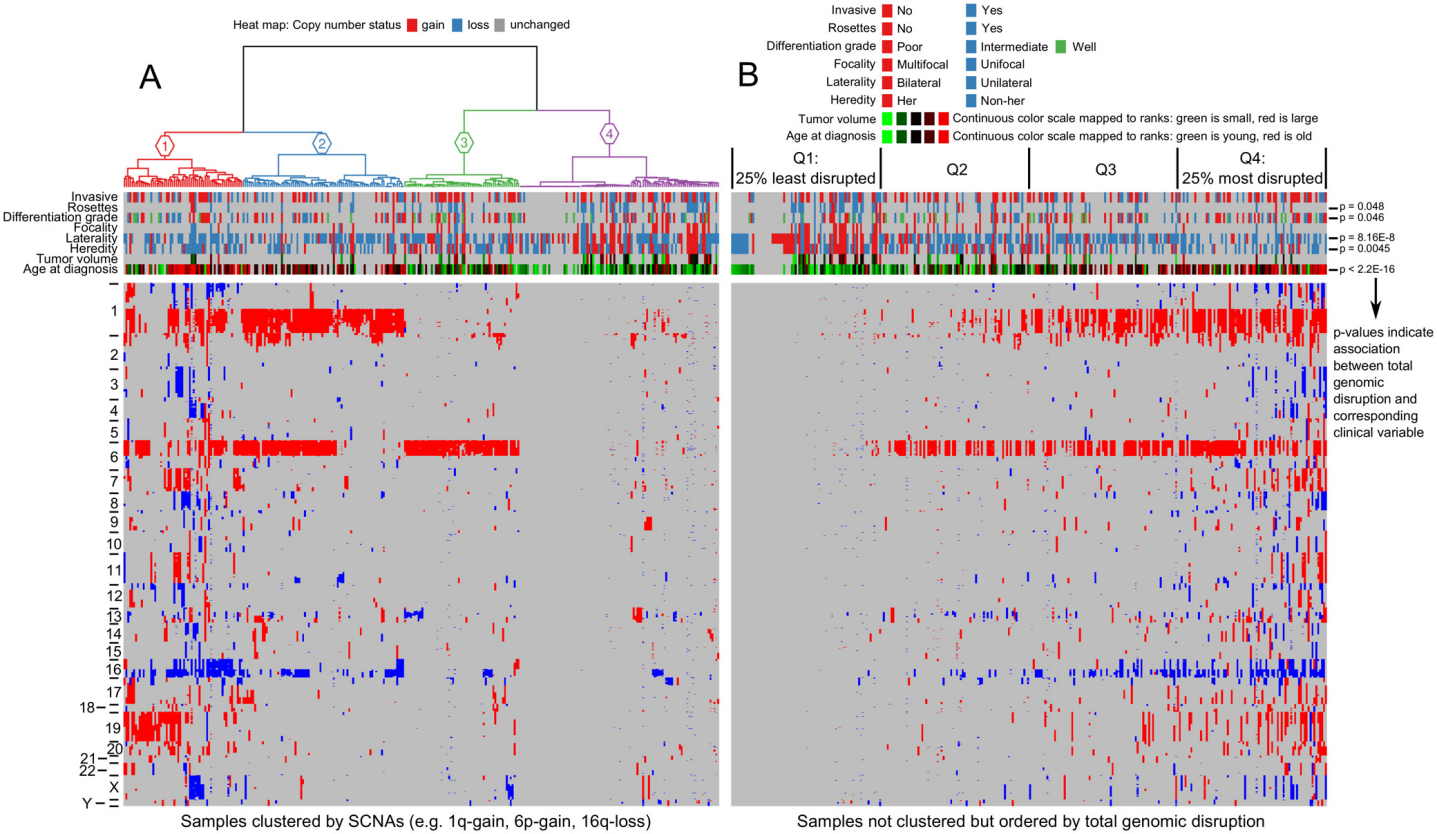
Clustering of genome-wide SCNA. Heat map of genome-wide SCNAs for 310 primary retinoblastoma samples (columns). Gains are indicated in red and losses in blue. (A) Samples are ordered by unsupervised hierarchical clustering (UHC) visualized by the dendrogram on top. The dendrogram was pruned in 4 UHC-groups indicated by UHC-group numbers and colored branches. Color-coded sample information: invasion: red = not-invasive, blue = invasive; rosettes: red = no rosettes, blue = rosettes; differentiation: red = poor, blue = moderate, green = well; focality: red = multifocal, blue = unifocal; laterality: red = bilateral, blue = unilateral; heredity: red = hereditary, blue = non-hereditary, tumor volume by MRI and age at diagnosis: continuous green-to-red scale where green means small tumors diagnosed at early age and vice versa. (B) Instead of ordering by UHC, samples are ordered based on total genomic disruption by SCNAs. P-values indicate the significance of association between total genomic disruption and the clinical variable.

Previous studies have shown that there is profound variability in copy number profiles of retinoblastoma samples associated significantly with age at diagnosis [7,8,12,15], heredity [15] and laterality [12,15]. In addition, profound differences in gene expression profiles between retinoblastoma samples were identified [19,20]. To identify genomic retinoblastoma subtypes, unsupervised hierarchical clustering (UHC) of retinoblastoma samples was performed using per gene SCNA-calls, displayed by a heat map (Fig 4A). The resulting retinoblastoma sample clustering is visualized by the dendrogram on top of the SCNA heat map together with corresponding color-coded sample information. The dendrogram was pruned to yield 4 UHC groups, optimizing for the differences between within cluster—and between cluster distances. Yet, it is arguable whether these 4 UHC groups represent truly distinct molecular retinoblastoma subtypes. To test for mutual exclusivity between frequently altered chromosomes, correlation tests were performed. None of the frequently altered chromosomes were significantly anti-correlated (S4 Fig). Instead of mutually exclusive SCNAs, clustering (Fig 4A) could be mainly driven by gradual variability in total genomic disruption. Indeed, total genomic disruption (in our study defined as the number of genes affected by SCNAs) was significantly different between the four UHC clusters. (Kruskal-Wallis p-value < 2.2E-16, Anova F-test: p-value < 2.2E-16). To further investigate a possible gradual variability in total genomic disruption, clustering (Fig 4A) was complemented by ordering the samples based on total genomic disruption (Fig 4B). There was a remarkable gradual increase in total genomic disruption that correlated with age at diagnosis (p-value < 2.2E-16). For each individual recurrent SCNA-gene, the frequency of occurrence increased with increasing total genomic disruption and age at diagnosis. Furthermore, recurrent 2p (mean age 25, standard error of the mean (SEM) 2.2 months) and 6p gains (mean 26, SEM 2.2 months) were observed in more stable and early diagnosed tumors (one-way ANOVA p-value 0.01) than 1q gains (mean 30 months, SEM 1.9 months) and 16q losses (mean 34, SEM 2.3 months). Possibly, total genomic disruption could be a better descriptive for SCNA-profiles than stratified groups identified by UHC.

### Genomic disruption increases with age at diagnosis, loss of differentiation, and SCNA-signal strength

**Fig 5:**
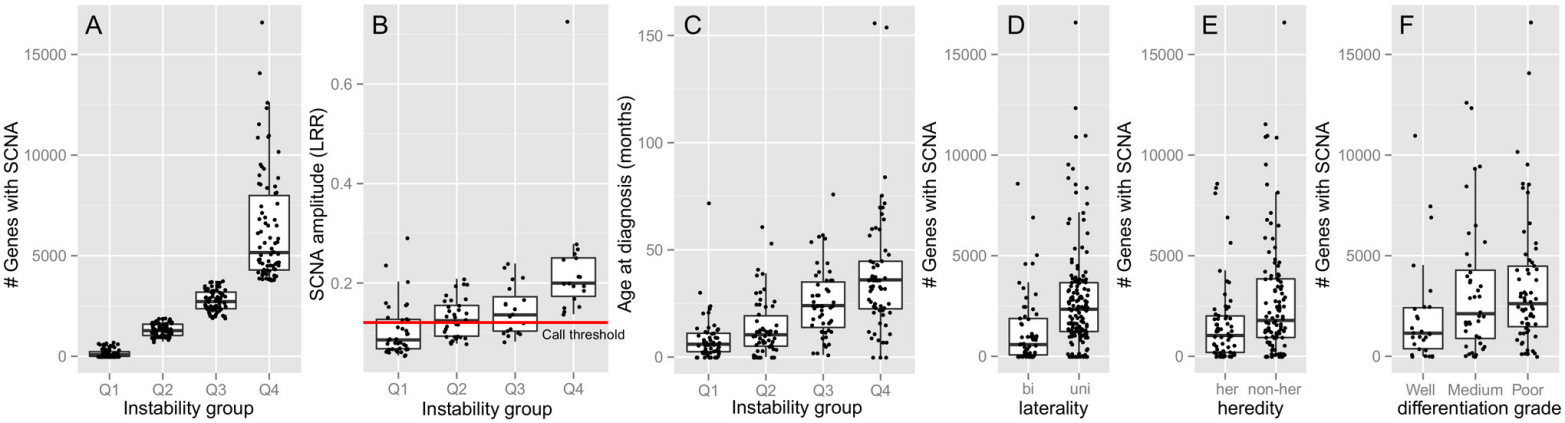
Relation of total genomic disruption with clinical data. Total genomic disruption by SCNAs with significantly correlated (p-values in Table 1) clinical and histopathological variables. Total genomic disruption is defined by the number of genes with either increased or decreased copy number. For numerical variables (B) age at diagnosis and (C) SCNA-amplitude, samples were categorized in quartiles based (A) on total genomic disruption. For example, Q1 denotes the 25% of tumors with the least genomic disruption and Q4 vice versa. SCNA-amplitudes are expressed as Log2-ratios. The horizontal red line illustrates the SCNA-threshold used for calling gains and losses (also see paragraph 2.4 in materials and methods and S6 Fig). For factorial variables (D) laterality, (E) heredity, and (F) differentiation grade the number of genes with SCNAs is stratified per factor level.

To assess the statistical significance of the association between total genomic disruption and clinical and histopathological variables, hypothesis testing was performed and presented in Table 1. Data of variables that significantly associated with total genomic disruption were also visualized (Fig 5). In case the clinical variable was numeric (e.g. age at diagnosis) the tumors were stratified in 4 disruption quartiles (Q1 = 25% least disrupted tumors, Q4 = 25% most disrupted tumors, Fig 4B) each showing boxplots of the clinical variable of interest. By definition, total genomic disruption differed significantly (p-value < 2.2e-16) between these disruption quartiles (Fig 5A). The average SCNA-amplitude per tumor was calculated from the segmentation mean of SCNAs with amplitudes both below and above the used segmentation threshold. Total genomic disruption linearly increased with SCNA-amplitudes (Fig 5B). This panel also shows that SCNA-amplitude rarely exceeds change of one copy (copy number 3, Log2-ratio 0.58). This means that there must be sample heterogeneity, either by intra-tumor clonal heterogeneity or contamination with non-cancer cells (e.g. retina or blood). For 40/66 (61%) samples in the Mol and our current study, DNA diagnostics identified LOH at the *RB1* allele as one of the disease causing events (S7 Table). For these tumors, the homozygosity at the *RB1* locus (mBAF values, S7 Table) is indicative for tumor cellularity. Tumor purity was estimated to be very high (mBAF > = 0.99) for 22/40 (55%) tumors, high (mBAF > = 0.90) for 14/40 (35%) and moderate (mBAF > = 0.74) for 4/38 tumors. Also in the tumors for which the tumor cellularity was estimated to be very high, the SCNA amplitudes rarely exceeded one copy. An example is given for tumor 101032–02 (S5 Fig) which clearly shows LOH at 13q and incomplete LOH at 16q. Since the tumor cellularity for the rest of the cohort (272/310, 88%) could not be determined, possible non-cancer cell contamination could not be ruled out for the majority of the cohort.

**Table 1:** Total genomic disruption in relation to clinical and histopathological variables.

Independent variable	Descriptive statistics	Hypothesis testing p-value
**Age at diagnosis (months)**	**Mean ± SEM**	<2.20E-016* (WRST)
Q1 (25% least disrupted tumors)	8 ± 1.4 months	
Q2	14 ± 1.8 months	
Q3	26 ± 2.6 months	
Q4 (25% most disrupted tumors)	38 ± 3.4 months	
**Laterality**	# genes with SCNAs:	8.16E-008* (WRST)
bilateral	1267 ± 230 genes	
unilateral	2845 ± 223 genes	
**Heredity**	# genes with SCNAs:	0.0045* (WRST)
hereditary	1618 ± 252 genes	
non-hereditary	2752 ± 285 genes	
**Differentiation grade**	# genes with SCNAs:	0.046* (LBL)
well	1998 ± 490 genes	
moderate	3307 ± 523 genes	
poor	3556 ± 420 genes	
**FW-rosettes**	# genes with SCNAs:	0.048 (WRST)
yes	1293 ± 327 genes	
no	2661 ± 844 genes	
**Focality**	# genes with SCNAs:	0.1144 (WRST)
multifocal	1016 ± 157 genes	
unifocal	2650 ± 658 genes	
**Volume measured by MRI (mm**^**3**^**)**	**Mean ± SEM**	0.1828 (WRST)
Q1 (25% least disrupted tumors)Q2	1028 ± 91 mm^3^976 ± 123 mm^3^	
Q3	773 ± 153 mm^3^	
Q4 (25% most disrupted tumors)	1120 ± 173 mm^3^	
**Sex**	# genes with SCNAs:	0.2191 (WRST)
male	2732 ± 278 genes	
female	2231 ± 228 genes	
**Rosettes**	# genes with SCNAs:	0.4004 (WRST)
yes	1325 ± 225 genes	
no	2303 ± 986 genes	
**Familiarity**	# genes with SCNAs:	0.4437 (WRST)
familial	1045 ± 651 genes	
non-familial	1507 ± 220 genes	
**Invasion**	# genes with SCNAs:	0.5951 (WRST)
invasive	3094 ± 326 genes	
non-invasive	2939 ± 457 genes	

Tested variables are sorted by statistical significance. Q1-4 denote cohort quartiles based on the number of genes whose copy number is altered where Q1 denotes the cohort quartile with the least copy number altered genes and Q4 the cohort quartile with the most copy number altered genes. WRST-Wilcoxon rank-sum test, LBL = Linear-by-linear.

* indicates significance (p-value < 0.05). P-values lower than 0.001 are given in scientific notation.

Possibly, the gradual differences in SCNA-amplitudes are caused by differences in within-tumor heterogeneity. It has been demonstrated before that within the same tumor, fields of SCNA-devoid differentiated benign precursor lesions were located adjacent to more undifferentiated malignant retinoblastoma fields full of SCNAs [5]. In agreement, increase in SCNA-amplitudes and total genomic disruption correlated with decreased differentiation grades (p-value 0.04).

### Retinoblastoma cell lines showed high total genomic disruption

**Fig 6:**
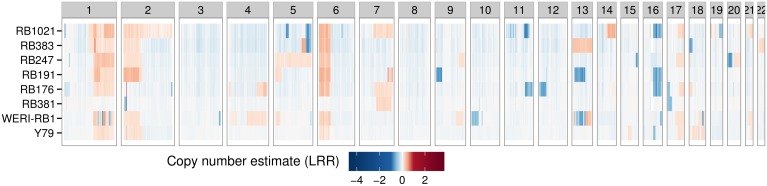
Copy numbers in retinoblastoma cell lines. Genome-wide heat map of SCNAs in 8 retinoblastoma cell lines. Sex chromosomes are not displayed since no matching reference was available. SCNA-amplitudes are expressed as Log2-ratio and mapped to a blue-to-red continuous color-scale.

Cell lines derived from retinoblastoma primary tissue are considered valuable model systems to study retinoblastoma *in vitro*. To assess the genomic resemblance of retinoblastoma cell lines to primary tumors, we determined genome-wide SCNA-profiles for 8 retinoblastoma cell lines and compared those to the 45 primary retinoblastoma samples. Furthermore, retinoblastoma cell cultures have been extensively selected for proliferation by *in vitro* culturing and might reveal focal SCNAs driving retinoblastoma proliferation that remained undiscovered in primary samples. Segmented SCNAs are visualized in a heat map for autosomal chromosomes per cell line (Fig 6). Chromosomes 1, 2 and 6 were gained in 7/8 cell lines (RB1021, RB383, RB247, RB191, RB176, WERI-RB1, and Y79) and chromosome 16q was (partially) lost in 5/8 (63%) cell lines (RB1021, RB191 and RB176, WERI-RB1, and Y79). Gain of chromosome 7 was observed in 4/8 (50%) cell lines (RB1021, RB191, RB176 and RB381) whereas cell line RB191 showed focal increase of copy number at 7q33 (135–136.5 Mb). This region includes 11 genes (*CNOT4*, *SDHDP2*, *NUP205*, *C7orf73*, *RNU6-1154P*, *SLC13A4*, *FAM180A*, *MTPN*, *LUZP6*, *RNU6-223P*, *and PSMC1P3)*. Only *NUP205* showed a significant association (FDR-adjusted p-value 2.62E-5) between copy numbers and expression and was also identified as a candidate in the primary tumor data set (S3 Table). Gain of chromosome 19q was not observed in any of the cell lines. The mean number of genes altered in cell lines (5813, S.E.M 1021) was significantly higher than in Q1 (p-value = 2.2E-06), Q2 (p-value = 3.7E-06) and Q3 (p-value = 1.3E-04), while not different from Q4 (p-value 0.65). This analysis indicates that retinoblastoma cell lines resemble primary tumors with high total genomic disruption or that they are a representation of the genomic disrupted part of the original tumors.

### Discussion

In our study, SCNA-profiles of 45 primary retinoblastoma samples were determined, which were analyzed together with SCNA profiles reported in ten published studies. In addition, the copy number data was integrated with publicly available matching gene expression data to further aid driver discovery. Candidate driver genes included *CRB1*, *NEK7* (1q), *MYCN* (2p), *SOX4* (6p), *RB1* (13q) and numerous genes for 16q. By dedicated subset analysis with the high-resolution platforms and by using alternative analysis algorithms, *MIR181* (1q) and *DEK* (6p) were identified additionally. Furthermore, our study shows examples of tumors with SCNAs do not exceed change of one copy, despite little non-cancer cell contamination, indicative for intra-tumor heterogeneity. Also, our meta-analysis allowed for a comprehensive association of retinoblastoma genotypes to the clinical phenotypes, which furthers our understanding of retinoblastoma.

### Candidate genes reported in previous studies

**Fig 7:**
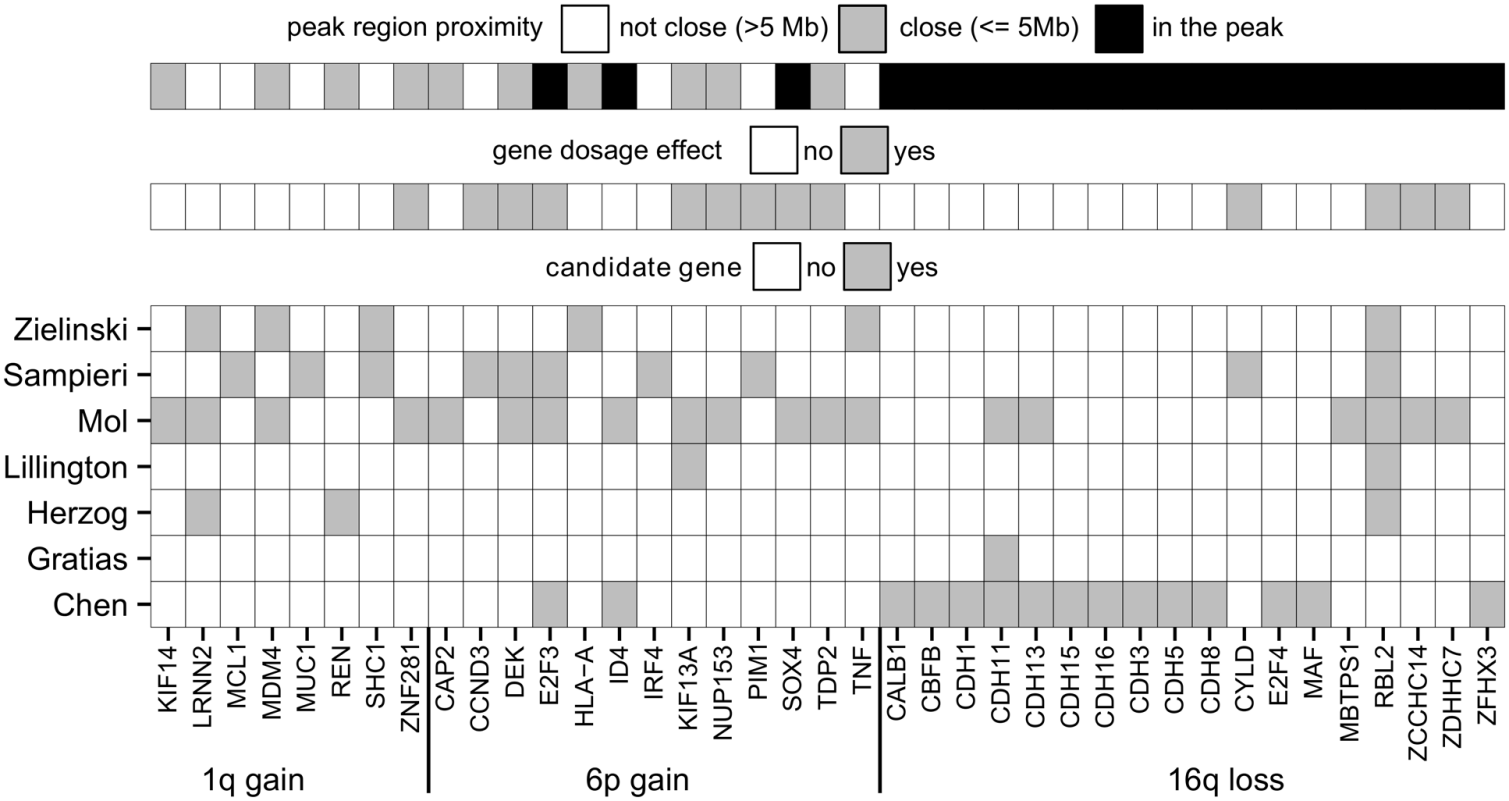
Reported candidate genes. Overview of putative candidate genes proposed by previous studies for commonly gained chromosomal arms 1q and 6p and commonly lost 16q.

Several studies aimed to identify genetic alterations promoting retinoblastoma development beyond loss of *RB1*. While most studies have focused on genetic alterations, it was also shown that epigenetic alterations might be important for retinoblastoma carcinogenesis [16]. Yet, the main focus of our current study is genetic alterations. Some of the previous retinoblastoma copy number alteration studies limited their discussion of SCNA-profiles to correlations with total genomic disruption (Mairal, van der Wal, Zhang), while other studies also provided suggestions for putative candidate genes beyond focally altered *MYCN* or *RB1* (Chen, Herzog, Lillington, Zielinski, Gratias, Sampieri, Mol). Although our study showed that the detected genome-wide SCNA-profiles showed good agreement between these studies (Fig 2), there was a clear variability in the suggested candidate genes between different studies (Fig 7).

### Candidate genes identified by the meta-analysis

For chromosome 1q-gains, eight different candidate genes have been proposed by previous studies. All of them are not located within the SCNA-gain candidate peak 1 region. Nevertheless, *KIF14*, *MDM4*, *REN*, and *ZNF281* were close (<5 Mb, see Fig 7, S5 Table) and *MDM4* and *REN* were located in the GISTIC peaks (S3 Fig, S5 and S6 Tables). In the meta-analysis, the most frequently gained genes with a significant gene dosage effect at chromosome 1q were *ZBTB41*, *CRB1* and *NEK7*. The *NEK7* gene showed the highest mean expression among these three candidates (S5 Table). The *NEK7* gene is part of the NIMA-related mitotic kinase gene family. In agreement, it was found that malignant retinoblastoma fields consist of mitotically active cells, in contrast to retinoma fields [21]. Also, overexpression of family member *NEK6* was shown to antagonize p53-induced senescence in human cancer cells [22]. Interestingly, benign precursor retinoma lesions stained positively for senescence-associated proteins p16^INK4A^ and p130 [5]. Possibly, gain of *NEK7* and subsequent protein overexpression can antagonize p53-induced cellular senescence and causes benign retinoma cells to progress though the cell cycle. Furthermore, the oncogenic potential of *NEK[6/7]* has been recognized in various cancers, including breast cancer [23], gallbladder cancer [24], Wilm's tumors [25] and head and neck cancers [26]. Using our integrative genome-wide approach, our study now also identified *NEK7* as novel 1q candidate gene potentially driving retinoblastoma progression.

Next to *NEK7*, the second most highly expressed gene in peak 1, *CRB1*, is a noteworthy candidate driver gene as well. *CRB1* is involved in the development of photoreceptor cells [27], which are suggested to be the cells of origin of retinoblastoma [28]. Expression of *CRB1* interrupts naturally occurring apoptosis and photoreceptor apoptosis required for proper retinal morphogenesis [29]. Mutations in the *CRB1* gene cause abnormally thick retina with abnormal lamination, in particular in the photoreceptor-dense are at the fovea [29]. Possibly, overexpression of *CRB1* driven by copy number gains accelerates photoreceptor-derived retinoblastoma.

Good concordance between our results and previously proposed candidate genes was observed for chromosome 6p gains. Out of 13 previously proposed candidates, *E2F3*, *ID4*, and *SOX4* are located in the candidate peak 2 region. For *ID4*, no significant gene dosage effect was observed in this study. To compensate for possible residual *RB1* activity, gain of *E2F3* might be important for retinoblastoma to develop. However, *SOX4* is also an interesting candidate gene. In hepatocellular carcinoma it was shown that *SOX4* over-expression led to a significant repression of p53-induced Bax expression and subsequent repression of p53-mediated apoptosis induced by gamma-irradiation [30]. Possibly, gain of *SOX4* in retinoblastoma could be a relevant hit beyond loss of *RB1*, allowing *RB1*-inactivated cells to better escape p53-induced apoptosis or senescence. Similarly to *NEK7*, *SOX4* has been identified as an important oncogene in a variety of other cancers including endometrial cancer [31], brain cancer [32,33], breast cancer [34], bladder cancer [35], ovarian cancer [36], colorectal cancer [37], liver cancer [30] and leukemia [38]. Therefore we suggest that *SOX4* should be considered as a serious candidate gene of the 6p gain region.

In case of chromosome 7q and 19p gains and 16q losses assignment of the driving gene is more speculative. Since focal SCNAs are not observed at these genomic loci, the resulting candidate peak regions contain numerous candidate genes. Several previous studies proposed *RBL2* (protein p130) as a candidate gene for the 16q loss (Fig 7) which is often seen in patients diagnosed at later age. Since p130 is primarily expressed in G_0_-cells restricting them for cell cycle entry [39], loss of p130 could prevent cellular senescence and promote the benign-to-malignant transition. Our study showed that copy numbers of *RBL2* are commonly reduced and are associated with decreased expression. Therefore, also in our analysis, it remains one of the many candidates for 16q loss. For chromosome 7q gains, out of 10 proposed candidates based on primary retinoblastoma samples, only *NUP205* is included in a focal gain observed in cell line RB191. In lung cancer cell lines it was shown that through TMEM209 stabilization of *NUP205*, protein levels of *MYC* were increased and promoted cell growth. Attenuation of TMEM209 stabilization corresponded to blocked growth, indicating the TMEM209-NUP250 complex might play a role in cell proliferation [40].

### Limitations of candidate gene identification

By integrating data from our DNA profiling study with published studies and integration with gene expression data, we present a comprehensive effort to identify retinoblastoma driving genes. A potential disadvantage of data pooling is that the resolution of the pooled data is lower than the resolution of the three high-resolution SNP-array studies. Another disadvantage is that SCNA amplitudes are not available for all studies. This meant that in the pooled analyses, SCNA amplitudes were not taken into account. Therefore, the pooled analysis was complemented by a subset GISTIC analysis using the high-resolution Mol, Zhang and Kooi datasets only (S3 Fig, S5 and S6 Tables). While GISTIC analysis confirmed the significance of 1q, 6p and 16q alterations and peak 2 (2p24.3, including *MYCN*) from the pooled analysis, the peak regions at 1q and 13q were slightly shifted in GISTIC analysis. For 1q, GISTIC even identified multiple regions to be significantly altered. Also when a different segmentation algorithm was used (genoCN, S2 Fig and S5 Table), the peaks at 1q and 6p slightly shifted, in this case towards the *MIR181* and *DEK* genes respectively. Except for 2p where only *MYCN* is included in all gained regions, multiple candidate genes remain for the commonly altered chromosomes. Therefore, functional assays are needed to empirically determine the oncogenic potential of the described candidate genes.

### Models explaining the association between total genomic disruption and age at diagnosis

Previous studies showed that retinoblastoma tumors have profound variability in total genomic disruption. It was unclear whether this variability is dichotomous or gradual, suggestive of two subtypes or gradual progression respectively. The study of van der Wal et al. suggested that variability in total genomic disruption was bimodal, although this was based on a small study (N = 13) and was not substantiated by any statistics. In the study of Mol et al. (N = 21), it was shown that unsupervised hierarchical clustering divided retinoblastoma samples into three branches with increasing total genomic disruption. Our study conclusively shows that total genomic disruption is gradual and co-occurrence and/or mutual exclusivity of SCNAs is not apparent. Increasing total genomic disruption was related to decreasing differentiation grades and suggests a de-differentiation process. Since our data includes indications for intra-tumor heterogeneity of genomic alterations, possibly there was also heterogeneity in differentiation grades between cells within tumors. In agreement, examples have been described where fields of differentiated cells lie adjacent to undifferentiated cells [21].

It is interesting why tumors with much genomic disruption and poorly differentiated cells were particularly observed in patients diagnosed at late age. Possibly, in tumors where the second *RB1* hit occurred at later age the resulting precursor lesion was less proliferative than lesions that developed more early in retina development. When SCNAs occurs in these late-onset precancerous lesions, the initial SCNA-devoid cells are easily overgrown by the progressed proliferative cells. The hypothesis that the proliferative consequence of *RB1* inactivation in the retina is age-dependent is underscored by the fact that retinoblastoma does not occur after the retina is fully developed. Additionally, diagnosis could have been delayed in patients with older age at diagnosis and thereby allowed the tumors more time to acquire SCNAs and progress.

### In conclusion

Our integrated approach allowed us to refine and improve the lists of putative retinoblastoma driving genes. This limited set of genes can serve as leads for future studies on retinoblastoma progression and precision medicine. However, we also found that for at least a subset of tumors, abnormal gene copy numbers were not always present in all tumor cells. Therefore, a multi-target treatment strategy might be required for efficient retinoblastoma treatment.

## Materials and Methods

### Tissue collection

Tumor samples were obtained from retinoblastoma patients after primary enucleation and peripheral blood samples were collected at initial presentation before treatment. In the Netherlands, all patients are referred to the VU University Medical Center. Hence a well-documented cohort of unselected primary enucleated eyes was available for molecular studies. Tumor samples were snap frozen in liquid nitrogen and stored at -80°C until further analysis. All patient samples and clinical and histopathological features were collected and stored according to local ethical regulations. All patients gave consent verbally, as this was the standard in the time the included patients were diagnosed. Since genetic analyses of our study focused on tumor DNA and not germ line DNA, waiver of informed consent was specifically given for genetic analyses by The Medical Ethics Review Committee of the VU University Medical Center which is registered with the USA OHRP as IRB00002991. The FWA number assigned to VU University Medical Center is FWA00017598. A cohort description including clinical and histopathological information is given in S2 Table. RB cell lines RB1021, RB383, and RB247 [41] were kindly provided by the laboratory of Brenda Gallie and cell lines RB191, RB176, and RB381 [42] by the laboratory of David Cobrinik.

### DNA isolation

Genomic DNA from frozen tumor retinoblastoma specimens was isolated with the NucleoSpin Tissue kit (Macherey-Nagel, Düren, Germany) or Wizard Genomic DNA Purification Kit (Promega, Madison, USA). DNA quality was analyzed for high molecular bands >20 Kb by agarose gel electrophoresis. DNA concentration and OD 260/280 ratio was determined with the Nanodrop ND-1000 spectrophotometer (NanoDrop Technologies, Wilmington, USA). DNA yields and quality were within the same range for all samples.

### SNP-arrays

Microarray-based DNA genotyping experiments were performed at ServiceXS (ServiceXS B.V., Leiden, The Netherlands) using the HumanOmni1-Quad BeadChip (Illumina, San Diego, USA), according to the manufacturer’s instructions. The BeadChip images were scanned on the iScan system and the data was extracted into Illumina’s GenomeStudio software v2010.1. The software’s default settings were used with the cluster file as developed by Illumina for genotype calling. Resulting copy number estimates (Log2-ratios between tumor and matched blood) and B Allele frequencies were normalized with tQn normalization [43] and segmented with DNAcopy [44] with a minimum segment length of five markers. For loss of heterozygosity detection, segmentation of converted B allele frequencies (mBAF) was performed using BAFsegmentation using a mBAF threshold of 0.8 [45]. A minimum of five consecutive markers was used for segmentation together with a minimum mBAF-amplitude of 0.6. In parallel to DNAcopy segmentation, genoCN segmentation was used with default parameters to infer copy number states gain, loss and unchanged of the SNP-array datasets. To identify significantly altered regions, GISTIC analysis was performed (q-value < 0.05) using the combined segmentation (by DNAcopy) results of the Mol, Zhang and Kooi data sets. Gene expression data is available at GSE59983 (primary samples) and GSE77094 (cell lines). DNA copy number data is available at EGAS00001001715.

### Data collection and analysis

By manual google scholar search, studies profiling SCNAs by CGH, array-CGH, SNP-array or NGS were identified (Fig 1). Studies that reported SCNAs by cytoband location were digitalized by manually looking up the current genomic coordinates of the reported cytobands (hg19). Careful examination of sample identifiers was performed to prevent duplicate records, yet no duplicate records were identified. In case SCNAs were reported in genomic coordinates, they were converted to hg19 coordinates by the UCSC liftover tool [46]. For studies where raw data was available [13,15,16], copy number estimates were segmented with DNAcopy as described above. Since biopsies are uncommon in retinoblastoma, the included samples are possibly biased for later staged tumors that had to be enucleated. Furthermore, earlier studies used lower resolution profiling compared to more recent studies and therefore more subtle genomic alterations might have been more readily identified by later studies.

All published SCNAs were concatenated with SCNAs detected by our study. SCNAs were called in three states; loss, normal and gain using copy number thresholds 1.8 (Log2-ratio = -0.15) for losses and 2.2 (Log2-ratio = 0.14) for gains for segments with a p-value < 0.05 (S6 Fig). For each official HGNC gene, the segmentation mean was calculated by overlapping genomic coordinates of the genes with detected SCNAs using BEDOPS [47]. For hierarchical clustering, Ward's agglomerative clustering was performed using Euclidean distances. Statistical analysis and visualization was done in R (Pumpkin Helmet, version 3.1.2). For hypothesis testing where both independent and dependent variables are numeric, Wilcoxon signed-rank tests were used. For 2-level categorical independent variables and numeric dependent variables, Wilcoxon rank-sum tests were used. For independent categorical variables with more than 2 unordered levels and numeric dependent variables, Kruskal-Wallis tests were used. For independent categorical variables with more than 2 ordered levels (e.g. differentiation grade low < medium < high) and dependent numerical variables, linear-by-linear association tests were used implemented by the “coin” R-package. Two-sided p-values below 0.05 were considered statistically significant. Extreme p-values lower than 2.2E-16 could not be calculated and are reported as <2.2E-16.

For the determination of gene-dosage effects, linear regression was performed of continuous copy number estimates (segmented Log2-ratios) on RMA-normalized expression estimates. The linear regression slope was tested for significance, and Benjamini-Hochberg multiple-testing corrected p-values were calculated. Genes with a positive regression slope (the more DNA copies, the more gene expression) and multiple-testing corrected p-values < 0.05 were considered to display a gene-dosage effect.

## Supporting Information

S1 Fig Determination of the SCNA percentage threshold. The number of genes remaining (Y-axis) after applying more stringent criteria for common events (X-axis, SCNA percentage threshold) rapidly decreases and reaches a transition point at SCNA percentage threshold between 5 and 10%. Our study defined SCNAs occuring in > 10% of the cohort to be frequent.
(TIFF)

S2 Fig Effect of Hidden Markov-Model segmentation. To determine the robustness of DNAcopy segmentation (Fig 2), genoCN segmentation implementing a Hidden Markov-Model approach was applied to the Mol, Zhang and Kooi studies (N = 111). For each gene, the frequency of gain and loss is given in the subset cohort.
(TIFF)

S3 Fig Subset analysis with GISTIC. Results of GISTIC analysis on the Mol, Zhang and Kooi dataset segmented by DNAcopy (N = 111). Regions with q-values below 0.05 were considered significant and are annotated with cytoband labels. Region coordinates and HGNC gene symbol annotations are provided in S6 Table.
(TIFF)

S4 Fig Correlations between SCNA peaks. Pearson correlation matrix testing for co-occurrence and mutual exclusivity between peak regions containing retinoblastoma-driving candidate genes. The lower-left triangle is a color-coded (blue = mutual exclusivity, red = co-occurrence) representation of the upper-right triangle which gives the Pearson correlations. Peak regions showed no mutual exclusivity and weak co-occurrence. The best correlation (0.42) was found between 1q gain and 16q loss, both events often observed in patients diagnosed at late age.
(TIFF)

S5 Fig Within-tumor heterogeneity of 16q-loss. An example of a tumor sample without non-cancer cell contamination (100% LOH at *RB1*, chromosome 13), but with incomplete LOH of 16q. Only SNPs that were heterozygous in the matching blood sample were used for this analysis. (A) Overview of mirrored B-allele frequencies (mBAF) segmented with BAFsegmentation. This sample displayed 100% LOH of 13q illustrated by mBAF ~ 1 indicating that this sample does not contain any detectable amounts of non-cancer cells. On the contrary, mBAF of 16q was segmented at mBAF 0.65, indicating that this sample contained cells with 16q-LOH (mBAF 1) and cells without 16q-LOH (mBAF 0.5). (B) B-allele frequencies of SNPs that were heterozygous in the matched germ line sample of chromosome 13 (complete LOH) and 16 (mixture of LOH and normal). Note that no data is available for the 13p-region since the DNA sequence of this region remains to be determined.
(TIFF)

S6 Fig Determination of gain and loss thresholds. Histograms of segmented Log2-ratios for Mol and Kooi (Illumina platform) and Zhang (Affymetrix platform) datasets. The dashed red and blue lines indicate the thresholds used for gains and losses respectively.
(TIFF)

S1 Table Description of SCNA studies. For each study included in the meta-analysis it is described how SCNAs were determined and integrated in our meta-analysis.
(XLSX)

S2 Table Cohort description. Statistics about the number of included samples per platform and patient phenotype variables.
(XLSX)

S3 Table Determination of candidate driver regions by meta-analysis. For chromosomes that were altered in at least 10% (31/310) of the pooled cohort, peak regions were identified. The peak regions were defined by the maximum SCNA gain-loss index for that chromosome with peak boundaries at 1% deflection from the peak. The peak regions were annotated with genes that displayed a gene-dosage effect and were considered candidate genes driving retinoblastoma oncogenesis.
(XLSX)

S4 Table Copy number and expression values. For 56 samples from the Mol and Kooi studies, both SNP-array and gene expression profiling was performed. For each gene that was profiled by both methods, the Log2-ratio (DNA) and Log2-transformed normalized expression (RNA) is given.
(XLSX)

S5 Table Per-gene SCNA-gain-loss values. For each approved HGNC gene, the SCNA-gain-loss difference is given together with gene dosage effect testing. In a subset analysis on the Mol, Zhang and Kooi dataset, the gain-loss difference was also determined using Hidden Markov-Model based segmentation (genoCN). Additionally, GISTIC analysis was performed in this subset and the per-gene q-values are given, indicating the per-gene significance of alterations.
(XLSX)

S6 Table GISTIC subset analysis results. Regions that were determined to be significantly (S3 Fig, q-value < 0.05) altered based on recurrence, amplitude and focality are given. Regions are annotated with HGNC gene symbols.
(XLSX)

S7 Table B-allele frequencies of *RB1*. For the Mol study and the current study, SNP-arrays were used and conventional DNA diagnostics for *RB1* was available. Using this data, the tumor cellularity can be estimated. In case the second hit was LOH (40 tumors), B allele frequencies of the *RB1* allele should often (34/40 tumors) exceeded 0.95 indicating non-cancer contamination at maximum was 10% for these tumors.
(XLSX)
