# Supplementary figures and images for: A Meta-Analysis of Retinoblastoma Copy Numbers Refines the List of Possible Driver Genes Involved in Tumor Progression
Source: PLoS One. 2016 Apr 26;11(4):e0153323. doi: 10.1371/journal.pone.0153323 (PMC4846005; doi:10.1371/journal.pone.0153323)

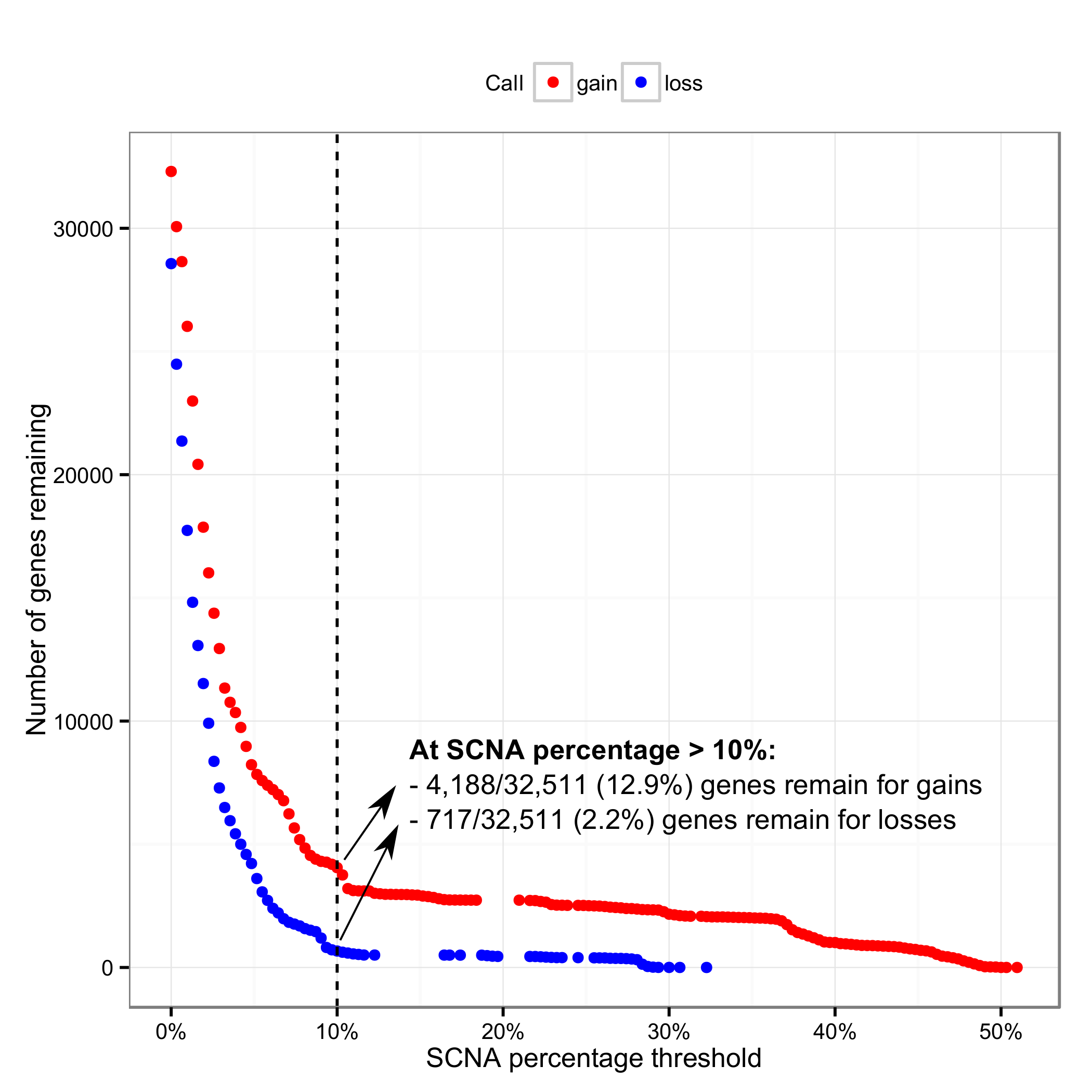

Supplement: S1 Fig — The number of genes remaining (Y-axis) after applying more stringent criteria for common events (X-axis, SCNA percentage threshold) rapidly decreases and reaches a transition point at SCNA percentage threshold between 5 and 10%. Our study defined SCNAs occuring in > 10% of the cohort to be frequent. (TIFF) [file pone.0153323.s001.tiff]

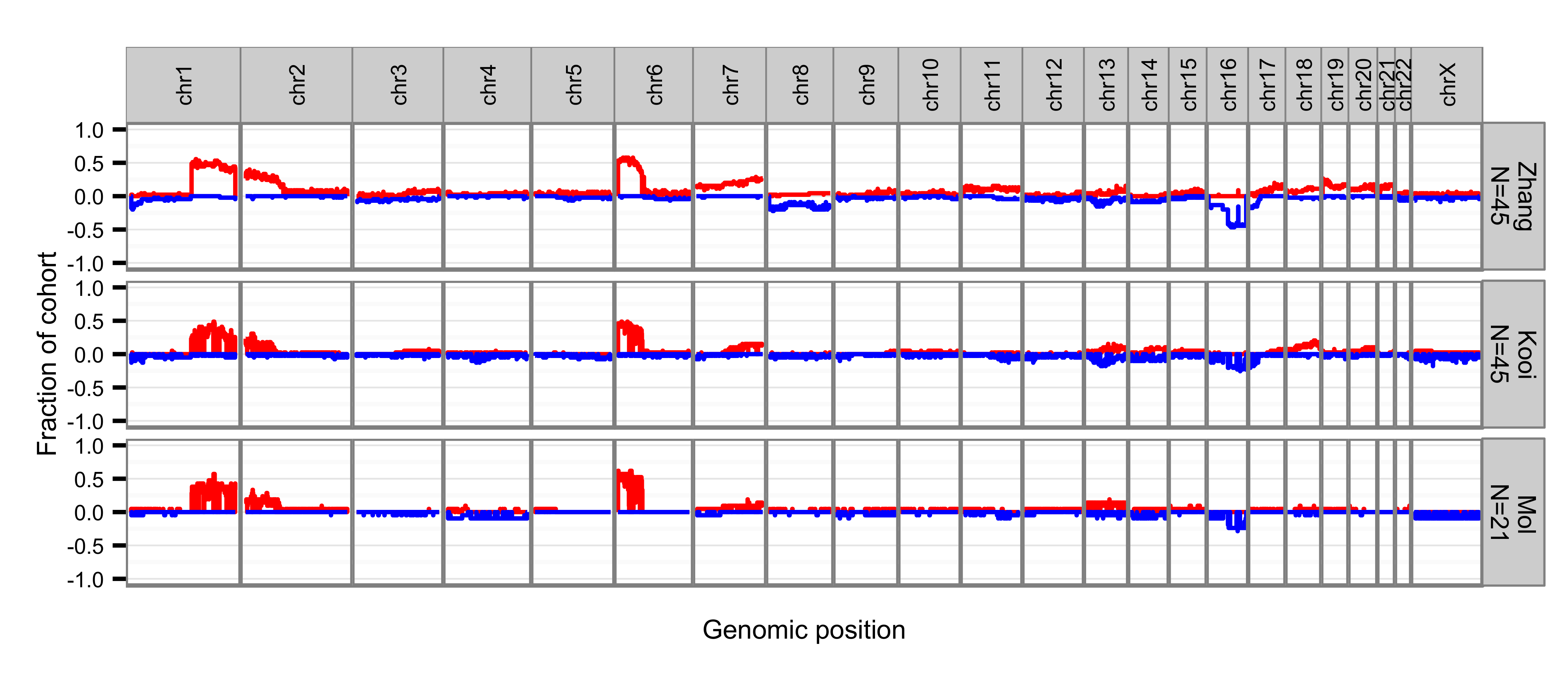

Supplement: S2 Fig — To determine the robustness of DNAcopy segmentation (Fig 2), genoCN segmentation implementing a Hidden Markov-Model approach was applied to the Mol, Zhang and Kooi studies (N = 111). For each gene, the frequency of gain and loss is given in the subset cohort. (TIFF) [file pone.0153323.s002.tiff]

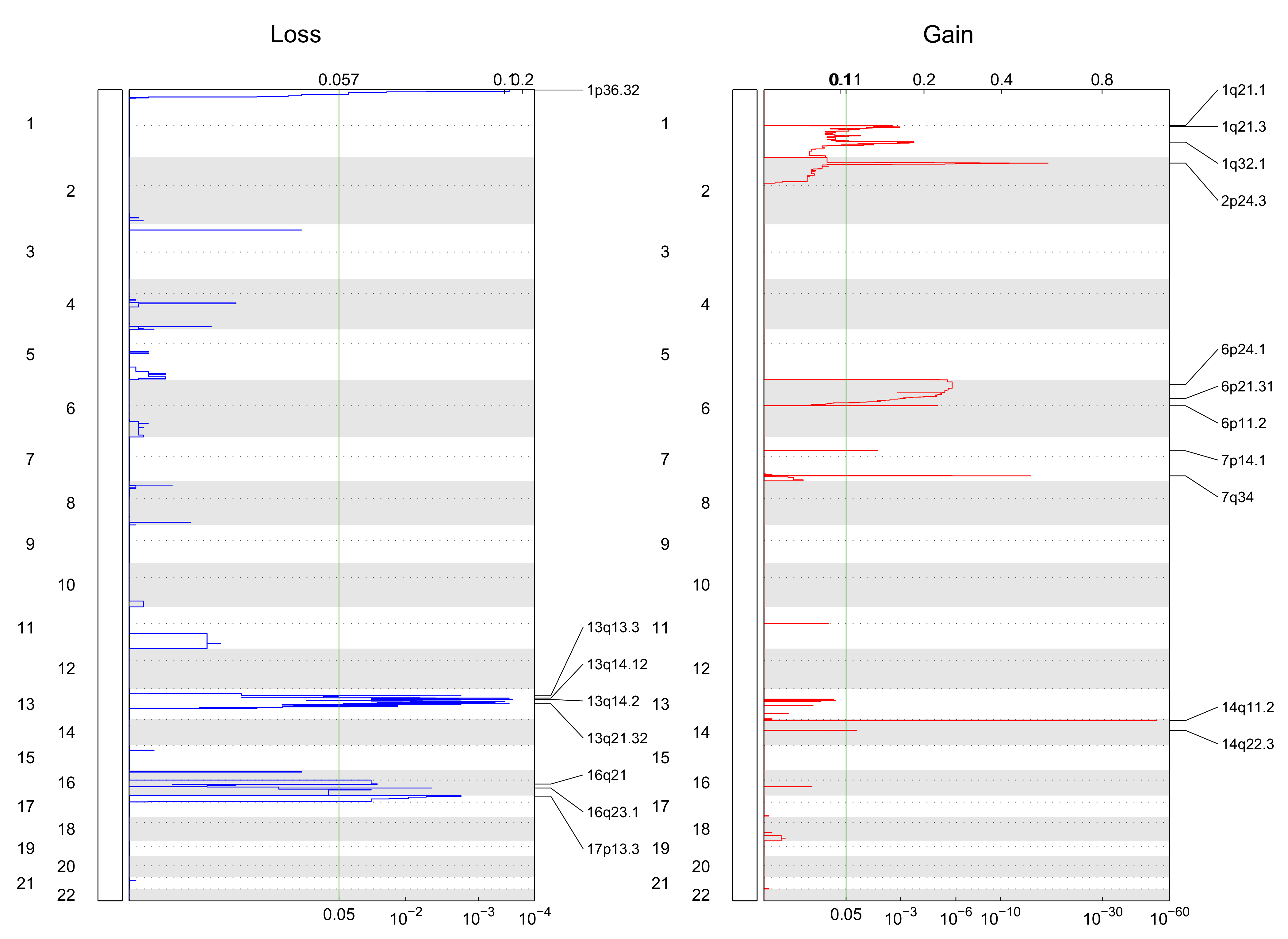

Supplement: S3 Fig — Results of GISTIC analysis on the Mol, Zhang and Kooi dataset segmented by DNAcopy (N = 111). Regions with q-values below 0.05 were considered significant and are annotated with cytoband labels. Region coordinates and HGNC gene symbol annotations are provided in S6 Table. (TIFF) [file pone.0153323.s003.tiff]

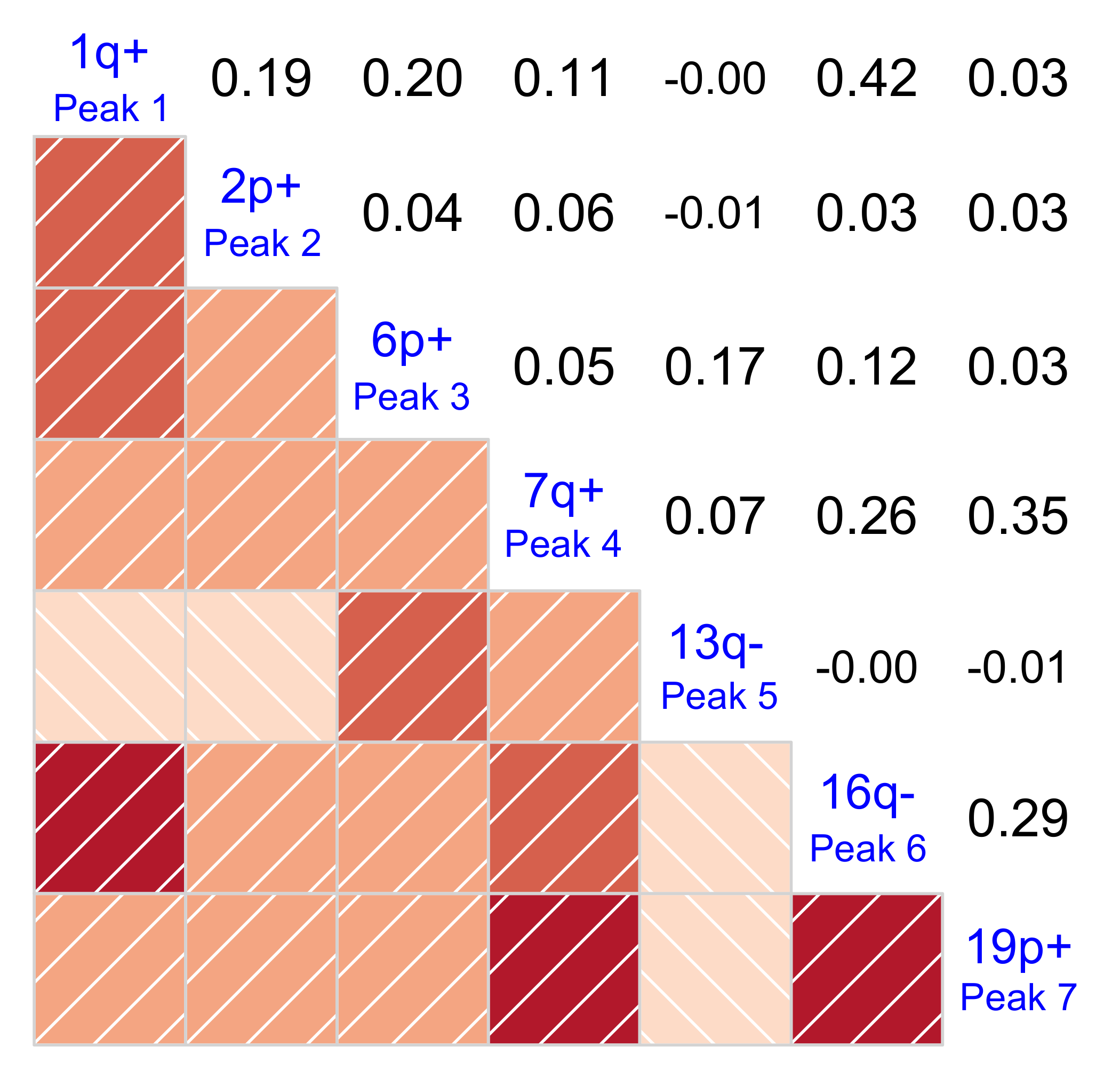

Supplement: S4 Fig — Pearson correlation matrix testing for co-occurrence and mutual exclusivity between peak regions containing retinoblastoma-driving candidate genes. The lower-left triangle is a color-coded (blue = mutual exclusivity, red = co-occurrence) representation of the upper-right triangle which gives the Pearson correlations. Peak regions showed no mutual exclusivity and weak co-occurrence. The best correlation (0.42) was found between 1q gain and 16q loss, both events often observed in patients diagnosed at late age. (TIFF) [file pone.0153323.s004.tiff]

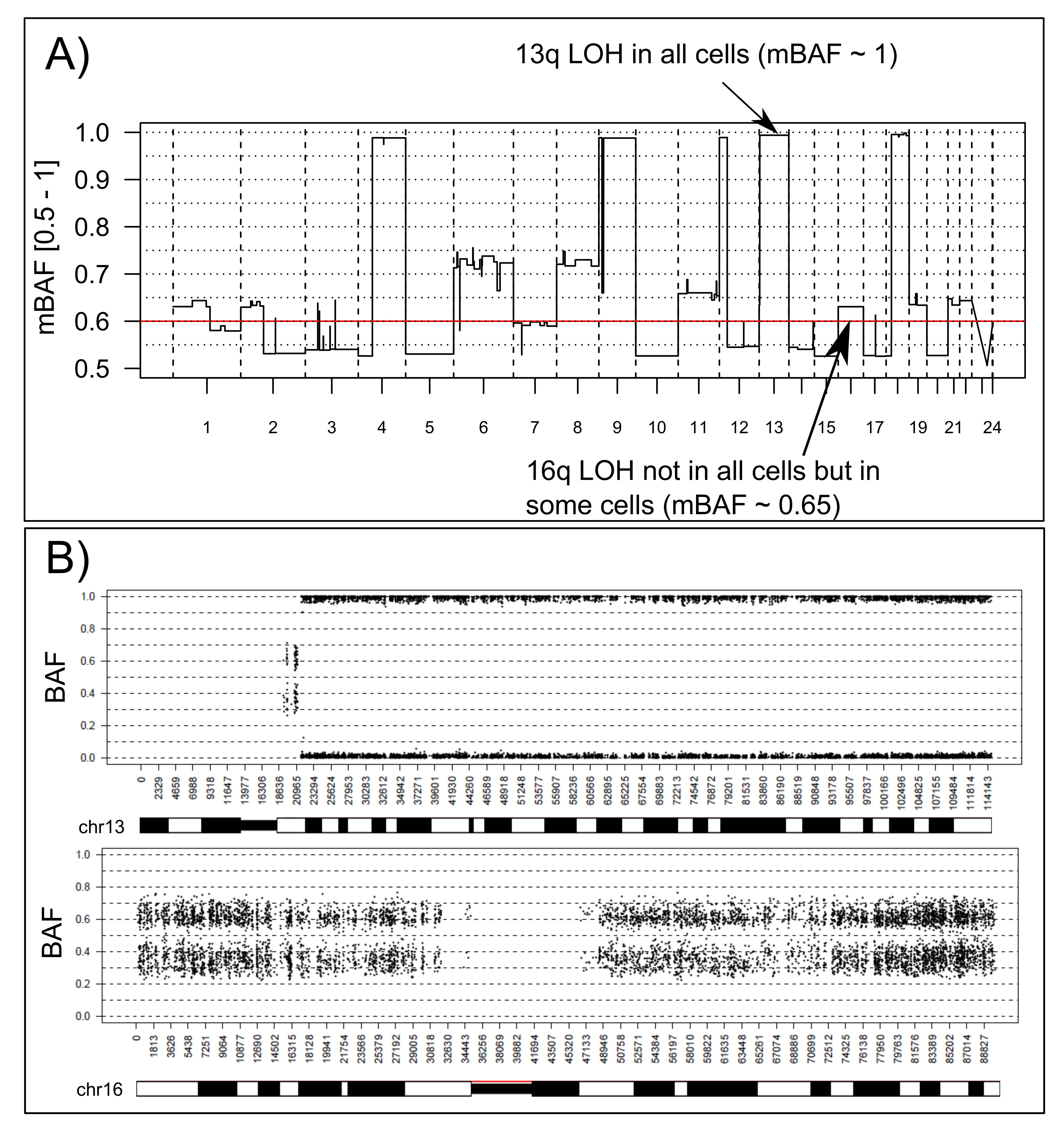

Supplement: S5 Fig — An example of a tumor sample without non-cancer cell contamination (100% LOH at RB1, chromosome 13), but with incomplete LOH of 16q. Only SNPs that were heterozygous in the matching blood sample were used for this analysis. (A) Overview of mirrored B-allele frequencies (mBAF) segmented with BAFsegmentation. This sample displayed 100% LOH of 13q illustrated by mBAF ~ 1 indicating that this sample does not contain any detectable amounts of non-cancer cells. On the contrary, mBAF of 16q was segmented at mBAF 0.65, indicating that this sample contained cells with 16q-LOH (mBAF 1) and cells without 16q-LOH (mBAF 0.5). (B) B-allele frequencies of SNPs that were heterozygous in the matched germ line sample of chromosome 13 (complete LOH) and 16 (mixture of LOH and normal). Note that no data is available for the 13p-region since the DNA sequence of this region remains to be determined. (TIFF) [file pone.0153323.s005.tiff]

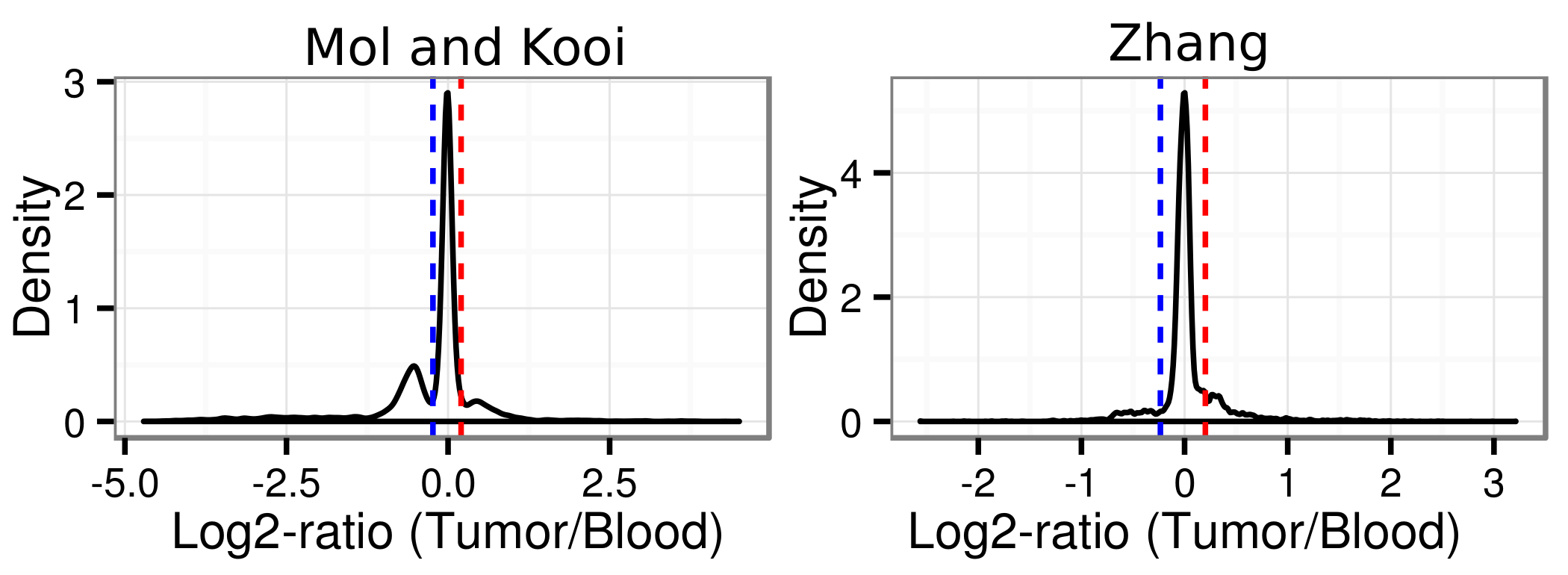

Supplement: S6 Fig — Histograms of segmented Log2-ratios for Mol and Kooi (Illumina platform) and Zhang (Affymetrix platform) datasets. The dashed red and blue lines indicate the thresholds used for gains and losses respectively. (TIFF) [file pone.0153323.s006.tiff]
